# De novo transcriptome assembly of the cotyledon of *Camellia oleifera* for discovery of genes regulating seed germination

**DOI:** 10.1186/s12870-022-03651-4

**Published:** 2022-05-28

**Authors:** Wei Long, Xiaohua Yao, Kailiang Wang, Yu Sheng, Leyan Lv

**Affiliations:** 1 Zhejiang Provincial Key Laboratory of Tree Breeding, Research Institute of Subtropical Forestry, Chinese Academy of Forestry, Hangzhou, 311400 Zhejiang China; 2 Department of Hydraulic Engineering, Zhejiang Tongji Vocational College of Science and Technology, Hangzhou, 311231 Zhejiang China

**Keywords:** *Camellia oleifera*, Cotyledon, Seed germination, Transcriptome, Hormone

## Abstract

**Background:**

*Camellia oleifera* (*C.oleifera*) is one of the most important wood oil species in the world. *C.oleifera* was propagated by nurse seedling grafting. Since the morphology of rootstocks has a significant impact on grafting efficiency and seedling quality, it is necessary to understand the molecular mechanism of morphogenesis for cultivating high-quality and controllable rootstocks. However, the genomic resource for this species is relatively limited, which hinders us from fully understanding the molecular mechanisms of seed germination in *C.oleifera*.

**Results:**

In this paper, using transcriptome sequencing, we measured the gene expression in the *C.oleifera* cotyledon in different stages of development and the global gene expression profiles. Approximately 45.4 gigabases (GB) of paired-end clean reads were assembled into 113,582 unigenes with an average length of 396 bp. Six public protein databases annotate 61.5% (68,217) of unigenes. We identified 11,391 differentially expressed genes (DEGs) throughout different stages of germination. Enrichment analysis revealed that DEGs were mainly involved in hormone signal transduction and starch sucrose metabolism pathways. The gravitropism regulator *UNE10*, the meristem regulators *STM, KNAT1*, *PLT2*, and root-specific transcription factor *WOX11* all have higher gene expression levels in the CAM2 stage (seed soaking), which indicates that the cotyledon-regulated program for germination had initiated when the seeds were imbibition. Our data showed differentially reprogrammed to multiple hormone-related genes in cotyledons during *C.oleifera* seed germination.

**Conclusion:**

Cotyledons play vital roles, both as the main nutrient provider and as one primary instructor for seed germination and seedling growth. Together, our study will significantly enrich the genomic resources of *Camellia* and help us understand the molecular mechanisms of the development in the seed germination and seedling growth of *C.oleifera*. It is helpful to culture standard and superior quality rootstock for *C.oleifera* breeding.

**Supplementary Information:**

The online version contains supplementary material available at 10.1186/s12870-022-03651-4.

## Background

The *C. oleifera*, one of the most important wood oil species in the world, is rich in unsaturated fatty acids and other nutrients, including vitamins, camelliaside, and tea polyphenols. It has been increasingly recognized in China because its seed oil has similar nutritional value as olive oil [1–3]. In breeding practice, the reproduction of *C.oleifera* depends on grafting, and the rootstocks required for grafting are derived from seedlings after seed germination. The growth of rootstocks has a significant impact on the grafting quality. Therefore, understanding the growth mechanism of rootstock seeds is essential for achieving efficient cultivation of rootstocks.

Seeds can sense and respond to environmental factors such as light [4], temperature [5], nutrients [6], and water [7], in order to control the precise timing of germination. “Once the embryonic growth potential exceeds the mechanical constraint of the surrounding tissues, including the cotyledon, germination is then complete.” [8]. This suggests that these environmental factors regulate common downstream events, probably targeting and acting on plant hormone metabolism and signaling. It has been shown that environmental signals can regulate hormone metabolism in the seed, as well as seed responsiveness to hormones [9–11]. Sand storage (low- temperature stratification treatment) is the most common method for ending seed dormancy. In the dormancy-breaking treatment, seeds are stored under wet conditions at 4℃.

Cotyledons play an important role in supporting embryonic growth by supplying nutrients, protecting the embryo, and controlling embryo growth by acting as a mechanical barrier during seed development and germination. Its structure and function in mature, dry seeds vary in different plant species. A subset of cotyledon tissues is composed of living cells after seed maturation, which play a critical role in regulating seed germination. As the nutrient source for seed germination, Cotyledons will participate in each stage of seedling morphogenesis after germination. So far, many studies have been conducted to examine endogenous hormones [12, 13], exogenous hormones, temperature, and other factors during seed germination of *C.oleifera* [14, 15], but molecular mechanisms of cotyledons participating in the morphogenesis of seedlings have been rarely investigated.

**Fig. 1:**
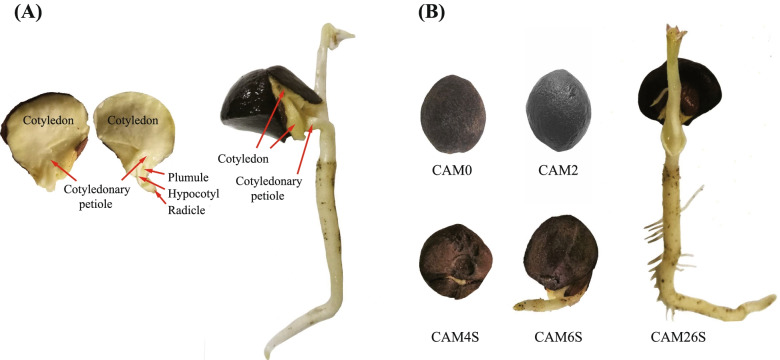
Characterization of *C.oleifera* seeds, and samples for RNA-seq. **A** Schematic illustration showing the structures including the plumule, cotyledonary petiole, hypocotyl, and huge cotyledon in seed of *C.oleifera*. **B** Seed or seedling of *C.oleifera* showing the cotyledon tissues (covered in brown seed coat) sampled for RNA-seq and hormone determination. CAM0: matured dry seed stored at 4℃, CAM2: imbibed seed soaked in purified water for 2 days, CAM4S: seed with the shell is broking, CAM6S: seed with the root growing to 2 cm in the sand stratification, CAM26S: seedling with the root growing to 10 cm in the sand stratification

In this paper, we explored the dynamic transcript profile of the functional transition of cotyledonary tissues during seed germination and seedling emergence of *C.oleifera* (Fig. 1), aiming to provide a clear understanding of the genetic elements that participate in this physiological process. The research can provide more accurate cultivation strategies and improve seedlings' propagation efficiency and quality, which ultimately helps achieve low-cost, high-efficiency, and automatic seedling breeding on *C.oleifera.*

## Results

### Illumina paired-end sequencing and de novo assembly, functional annotation

In total, we obtained 707,382,370 raw reads, from which a total of 690,806,162 clean reads were produced after quality control (Additional file 2: Table S1). All clean reads were deposited at NCBI and can be accessed via SRP349608. All of the high-quality reads from these fifteen libraries were mixed for transcriptome assembly using the Trinity software. All clean reads were de novo assembled into 284,782 transcripts with a mean length of 441 bp and N50 of 1,308 bp. These reads would be assembled into 113,582 unigenes, with a mean length of 396 bp and N50 of 934 bp (Additional file 3: Table S2). Based on homologous searches, 68,217 (60.06%) unigenes had achieved blast by DIAMOND program hits in at least one of the six databases (Additional file 4: Table S3). Further analysis of homologies had the highest homology with sequences from *Vitis vinfera* (19.25%), followed by *Oryza sativa* (5.71%), *Juglans regia* (4.51%) (Additional file 1: Fig. S1).

### Functional categories of the unigenes

**Fig. 2:**
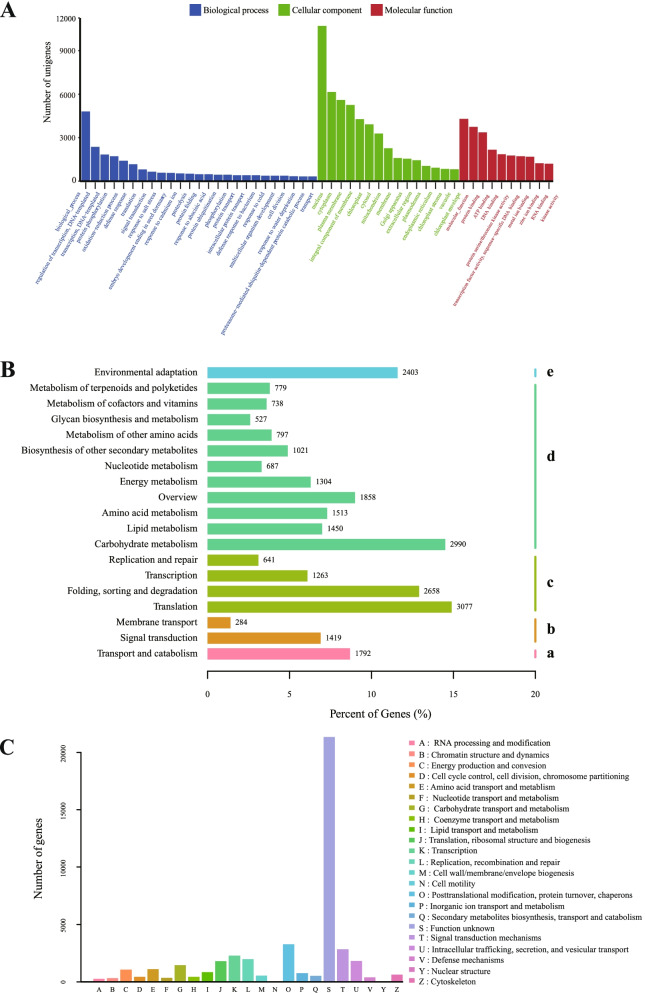
Functional categories of the unigenes. **A** Histogram presentation of GO classification. Bars represent the numbers of unigenes matched to each GO term of three categories: Biological process (blue), Cellular component (green). Molecular function (red). **B** The metabolism pathways are assigned into five categories. (a) Cellular processes. (b) Environmental information processing. (c) Genetic information processing. (d) Metabolism, and (e) Organismal systems. **C** Histogram presentation of eggNOG classification. Unigenes with significant matches in the NOG database were classified into 24 categories

GO classification and KEGG pathway-based analysis were performed to gain insight into the assembled unigenes' functional categorization.. A total of 38,657 unigenes were assigned to GO terms, which were classified into 50 functional groups under three principal categories, i.e., biological process, Molecular function, and Cellular components (Fig. 2A, Additional file 5: Table S4). In the Biological process category, the largest subgroups were “biological process”, “regulation of transcription, DNA templated”, and “transcription, DNA templated”; In the cellular components category, “nucleus”, “cytoplasm”, “plasma membrane” were the most highly represented ones. As for the Molecular function category, the most abundant genes were associated with “molecular function”, “protein binding”, and “ATP binding”.

Besides, a total of 20,652 unigenes were matched in the KEGG database and assigned to 138 pathways in five KEGG biochemical pathways, including “metabolism”, “Genetic information processing”, “Organismal systems”, “Cellular processes”, and “Environmental information processing” (Fig. 2B, Additional file 5: Table S4). The three most represented pathways were “translation”, “carbohydrate metabolism”, and “folding, sorting, and degradation”, followed by “environment adaptation” and “overviews”, whereas “membrane transport” and “replication and repair” pathways represented the smallest categories. The pathways are associated with seed germination, including “plant hormone signal transduction”, “starch, and sucrose metabolism”, and “biosynthesis of amino acids”.

All the assembled unigenes were subjected to a search against the evolutionary genealogy of genes to evaluate the annotation's effectiveness and the transcriptome library's completeness: Non-supervised Orthologous Groups (eggNOG) database. Unigenes were assigned into 23 categories (Fig. 2C, Additional file 6: Table S5). The top three categories were “function unknown”, “posttranslational modification, protein turnover, chaperones”, and “signal transduction mechanisms”, followed by “transcription”, and “replication, recombination and repair”; The smallest category was “cell motility” and “nuclear structure”.

### Identification and functional classification of DEGs

**Fig. 3:**
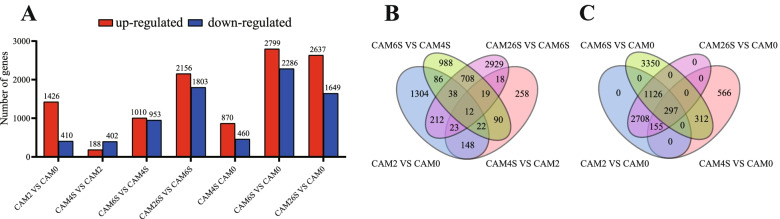
Comparative analysis of tissue-enriched and differentially expressed genes. **A** Histogram shows the distribution of different genes in adjacent stages and different stages compared to CAM0. **B** Venn diagram showing the genes in tissue compared to adajacent stages. **C** Venn diagram showing the genes in tissues compared to CAM0

The fifteen samples were separated into five groups in the principal component analysis (PCA) plot, and three replicates of each sample were grouped. Three seed samples at different developmental stages were closer to each other than to other samples (Additional file 1: Fig. S2). DEGs during seed germination were identified by pairwise comparisons at different time points to find germination-induced genes in seeds. The number of DEGs in CAM2 vs. CAM4S was minimum, and there were much more DEGs in CAM2 vs. CAM6S and CAM6S vs. CAM26S. Thus, most gene expression changes occurred after the water uptake stage (CAM4S) (Fig. 3A, Additional file 7: Table S6). A Venn analysis showed the distribution of common and unique DEGs between different adjacent stages and pairs of CAM0 vs. CAM2, CAM0 vs. CAM4S, CAM0 vs. CAM6S, and CAM0 vs. CAM26S. There were 12 common DEGs in adjacent stages (Fig. 3B, Additional file 8: Table S7). Comparatively speaking, there were more common DEGs (297) in pairs of CAM0 vs. CAM2, CAM0 vs. CAM4S, CAM0 vs. CAM6S, and CAM0 vs. CAM26S (Fig. 3C, Additional file 8: Table S7), indicating that the gene expression was substantially altered throughout different seed germination stages of *C.oleifera*. These phase-related gene expression changes during seed germination may have important functional implications for the growth of *C.oleifera* seed.

To gain insights into the functional categorization and metabolic pathways of DEGs involved in seed germination, 6820 of the 11,391 DEGs in cotyledons during *C.oleifera* seed germination were subjected to function annotation and function enrichment analysis. Abundant GO terms like “nucleus”, “transcription, DNA-templated”, “molecular function”, “zinc ion binding”, “plasma membrane, and “protein serine/threonine kinase activity” were enriched in adjacent stages, and pair of CAM0 vs. CAM2, CAM0 vs. CAM4S, CAM0 vs. CAM6S, and CAM0 vs. CAM26S (Fig. S3, Additional file 9: Table S8). In addition, the results of the 3810 DEGs mapped to the KEGG database showed that a large number of genes were involved in the pathways related to “plant-pathogen interaction”, “carbon metabolism”, “endocytosis”, “ribosome”, “RNA transport”, “Protein processing in endoplasmic reticulum”, “spliceosome”, “starch and sucrose metabolism”, “phenylpropanoid biosynthesis”, and “plant hormone signal transduction” (Fig. S4, Additional file 9: Table S8). This suggests a crucial role of hormones regulating *C.oleifera* seed germination.

Besides, the GO terms of 12 common DEGs among adjacent stages mainly included plasma membrane, dioxygenase activity, and nucleosome. The GO terms of 297 common DEGs with pairs of CAM0 vs. CAM2, CAM0 vs. CAM4S, CAM0 vs. CAM6S, and CAM0 vs. CAM26S, mainly included “nucleosome, “molecular function”, “secondary metabolic biosynthesis pathway”, “signal transduction”, and “vacuolar membrane”. The genes involved in the KEGG pathways were related to the mRNA surveillance pathway, including “cyanoamino acid metabolism”, “plant hormone signal transmission”, “endocytosis”, “plant pathogen interaction”, “protein processing in endoplasmic reticulum”, and “2-oxocarboxylic acid metabolism” (Additional file 8: Table S7).

### Identification of genes associated with hormones signaling in seed germination

**Fig. 4:**
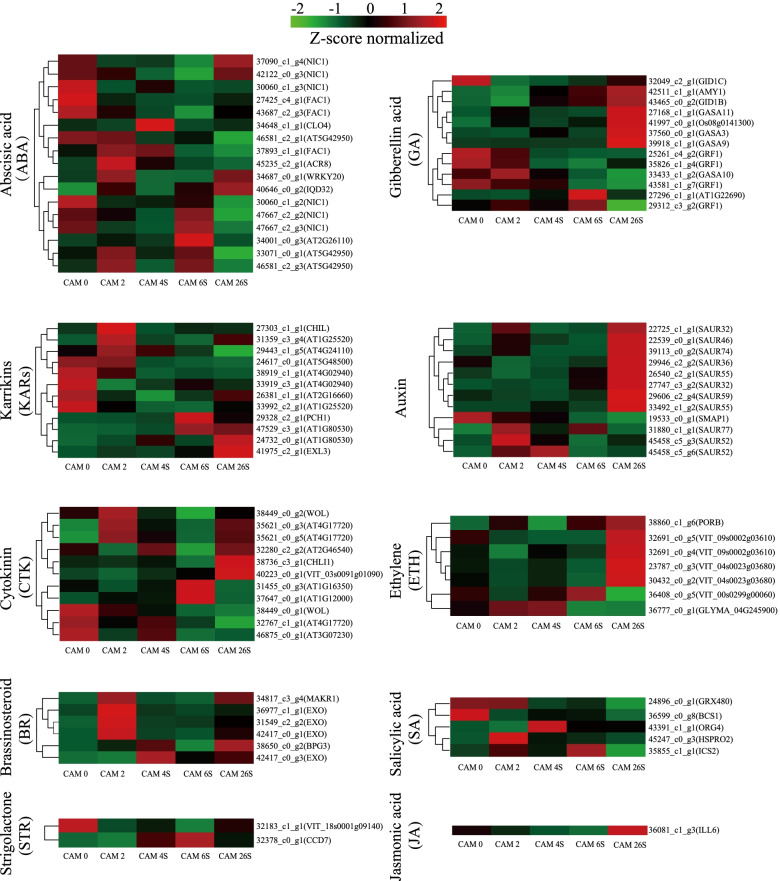
Expression profiles of genes associated with hormones signaling across five different stages. The expression of genes involved in hormones signaling and transduciton-related pathways was Z-score normalized and hierarchically clustered in heatmap. Z-scores were calculated from the log2 fold change values. Z-score = (log2 fold change of TPM values—mean of log2 fold changes of all TPM values of the samples) / standard deviation of log2 fold changes of all TPM value of the samples. A color scale is showed at the top. Green color indicates lower expression, and red color indicates higher expression

To gain insights into the functional roles of hormones during the seed germination *of C.oleifera*, we mapped the DEGs to hormone signaling and transduction pathways and analyzed their expression in different stages (Fig. 4). A total of 86 genes were identified to be associated with the biosynthesis, metabolism, and signaling of ten hormones, including Abscisic acid (ABA), Auxin (IAA), Ethylene (ETH), Karrikins (KARs), Brassinosteroid (BR), Cytokinin (CTK), Gibberellin acid (GAs), Strigolactone (STR), Salicylic acid (SA) and Jasmonic acid (JA). The genes associated with ABA biosynthesis and metabolism were chosen as the largest group with 17 members, followed by GAs with 13 members, KARs and Auxin with 12 members, and CTK with 10 members. In contrast, the genes with JA signaling belonged to the smallest group with only 1 member. These hormone-signaling and transduction-related genes exhibited differential expression across the different examined stages. A significant proportion of DEGs related to GA, Auxin, and ETH had high expression levels in the CAM26S stage, indicative of their potential roles in seedling growth. Besides, most of the genes that respond to KARs and BR are relatively highly expressed at CAM0 and/or CAM2 stage, suggesting that they may function in the early stage of seed germination.

### Identification of transcription factors in *C.oleifera*

**Fig. 5:**
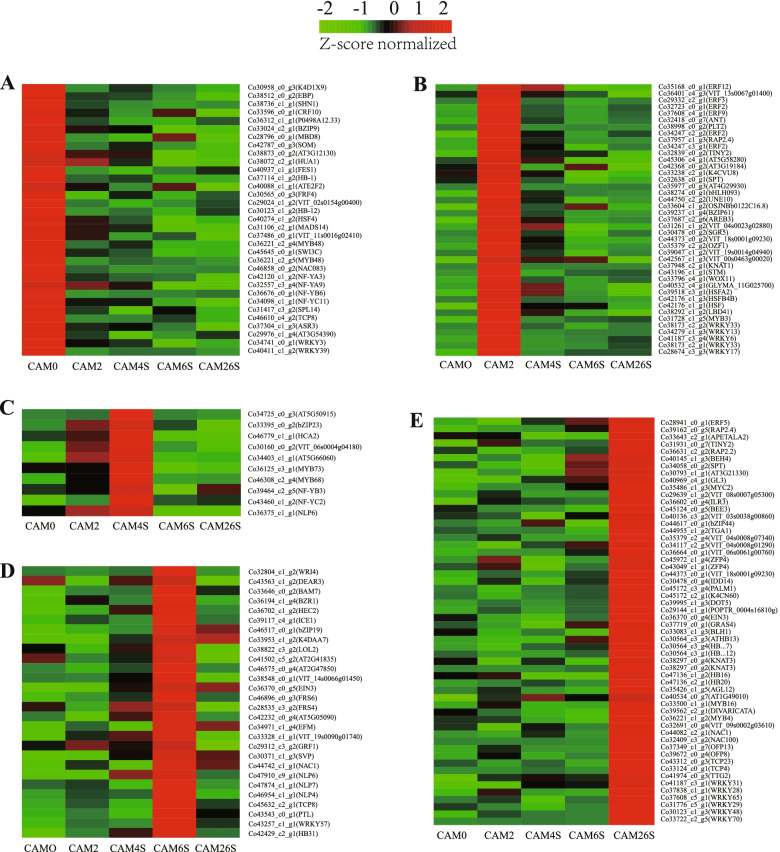
Relatively highly expressed TF-encoding genes identified at specific stages. **A** Heatmap displaying the special expression patterns of TFs in samples of CAM0; **B** Heatmap displaying the special expression patterns of TFs in samples of CAM2; **C** Heatmap displaying the special expression patterns of TFs in samples of CAM4S; **D** Heatmap displaying the special expression patterns of TFs in samples of CAM6S; **E** Heatmap displaying the special expression patterns of TFs in samples of CAM26S

Transcription factors (TFs) play critical roles in various plant development processes. To provide insights into the regulatory network underlying seed germination, we examined the expression of TFs, especially their dynamic differential expression. 310 TFs belonging to 36 different families were found to be differentially expressed in the stages examined**.** The TFs displayed expression patterns across the five stages. A majority of TFs showed relatively broad expression patterns in all stages (Additional file 1: Fig. S5), while some exhibited distinctive stage-specific patterns (Fig. 5). Interestingly, multiple transcription factor members of the WRKY and ERF family, which respond to GA signals [16], were significantly highly expressed in the CAM2 stage (soaking seeds). In addition, the meristem regulator *SHOOTMERISTEMLESS* (*STM*), *KNOTTED- like from Arabidopsis thaliana* (*KNAT1*), *PLETHORA* 2 (*PLT2*), and *WOX11*, as the root-specific transcription factor, was also observed to be highly expressed in this stage [17, 18].

### Co-expression network analysis with WGCNA

**Fig. 6:**
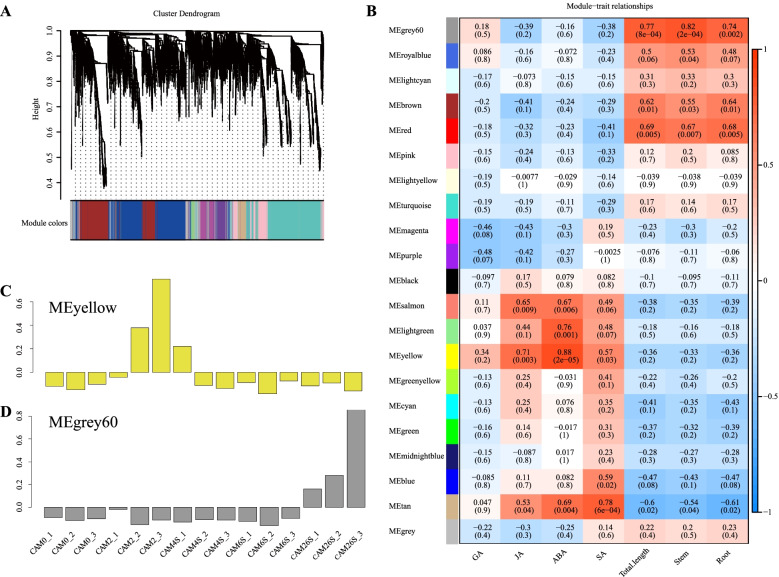
Weighted Gene Co-expression Network Analysis (WGCNA) of the unigenes change in *C.oleifera.* **A** Hierarchical cluster dendrogram using unigenes identified by WGCNA in different germination stages across 20 co-expression modules. **B** Module-tissue associations. Each row corresponds module. Each column corresponds to a specific tissue. The color of each cell at the row-column intersection indicates the correlation coefficient between the module and the developmental stage. A high degree of correlation between a specific module and the development stage is indicated by the red or blue color. **C** The gene expression patterns in MEyellow module. **D** The gene expression patterns in MEgrey60 module

To identify the clusters of highly interconnected genes that were specific to tissues, co-expression networks were constructed on the basis of pairwise correlations between genes in their common expression trends across all sampled tissues. As observed in the dendrogram, 20 unique modules of eigengenes have been identified (Fig. 6A, Additional file 10: Table S9). Notably, 4 out of 20 co-expression modules consist of genes that are significantly relevant to hormones and growth during seed germination (*r* > 0.7, *P* < 10^–2^) (Fig. 6B).

Here, two modules (MEyellow and MEgrey60) were listed for further analysis. The MEyellow module, including 935 genes (Additional file 10: Table S9), was highly correlated with the content of ABA **(**Fig. 6B, C**)**. According to the GO analysis, all genes in the MEyellow module were highly enriched in molecular function, zinc ion binding, quercetin 7–0-glucosyltransferase activity, transcription factor activity, UDP-glycosyltransferase activity, and sugar: proton symporter activity, etc. (Additional file 7: Table S6). Those enriched in the KEGG pathways were associated with plant-pathogen interaction, plant hormone signal transduction, circadian rhythm-plant, amino sugar, and nucleotide sugar metabolism, etc. (Additional file 10: Table S9).

WGCNA can also be employed to construct gene networks, in which each node represents a gene and the connecting lines (edges) between genes represent co-expression correlations. Hub genes refer to those that show most connections in the network. In the MEyellow module network, there were 27 genes that encode transcription factors and 4 hormone-related genes. Most of these transcription factor genes were highly expressed during seed soaking and may regulate or participate in activities operating in seed germination (Additional file 10: Table S9).

**Fig. 7:**
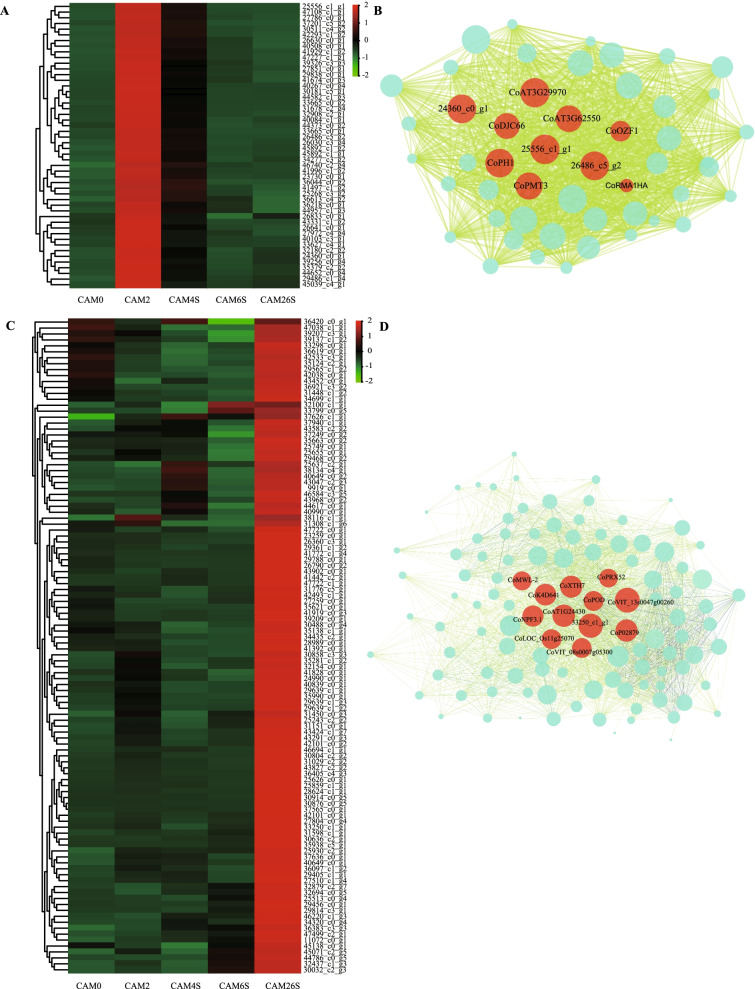
Analysis of genes in MEyellow and MEgrey60 modules. **A** The heat map of the most highly connected 50 hub genes in the MEyellow module. **B** Co-expression networks of the most highly connected 50 hub genes in the MEyellow module. **C** The heat map shows the relative TPM of each gene in the MEgrey60 module. **D** Coexpression networks of the most highly connected hub genes in the MEgrey60 module

The 50 most highly connected hub genes in the MEyellow module were used for analyzing the gene expression network. Gene expression showed that the expression level in the stage of seed soaking was higher than that in other stages (Fig. 6B, 7A Additional file 11: Table S10). Co-expression network showed core hub genes, namely, 25556_c1_g1 and 26030_c3_g4. The function of the 25556_c1_g1 gene remains unknown. The 26030_c3_g4(*AT3G29970*) gene belongs to molecular function. Other highly connected hub genes include signal transduction *CoPH1*(*25268_c3_g2*), sugar:proton symporter activity *CoPMT3*(*26833_c0_g1*), vacuole *CoAT3G62550*(*27851_c0_g1*), and *CoDJC66*(*29838_c0_g1*) (Fig. 7B). Interestingly, *CoPMT3* (*26833_c0_g1*) is annotated into the protein O-mannosyl transferase gene family (PMT), which is an important sugar proton symporter activity, and produces small ubiquitin-like modifiers (SUMOs). PMTs have differential tissue-specific functions in phosphatidylcholine (PC) biosynthesis and plant growth. As primary enzymes for phosphocholine (PCho) biosynthesis, *PMT3* are involved in PtdCho biosynthesis and vascular development in *Arabidopsis* seedlings [19, 20].

The 110 genes in the MEgrey60 module were highly correlated with the growth of seedlings (Fig. 6B, 6D). These genes were highly enriched in GO terms, including molecular function, protein serine/threonine kinase activity, transferase activity, sugar: proton symporter activity and transcription factor, secondary metabolite biosynthetic process, signal transduction, transcription, and transmembrane transport (Additional file 10: Table S9). Besides, KEGG analysis showed that those genes were enriched in pathways associated with plant-pathogen interaction, plant hormone signal transduction, phenylpropanoid biosythesis, cyanoamino acid metabolism, limonene and pinene degradation, and brassinosteroid biosynthesis (Additional file 10: Table S9**)**. The hub genes with the maximum number of edges (81) is *CoVIT_13s0047g00260* (28624_c1_g1), a member of the *shiu*: CAMK1 protein kinase family, which is associated with protein autophosphorylation, transporter activity [*CoAT1G24430* (29788_c0_g1), *CoNPF3.1* (40839_c0_g1), *CoK4D641*(gene43291_c0_g3)], xyloglucan: xyloglucosyl transferase activity [*CoXTH7* (30876_c0_g5)], protein binding [*CoP02879*(41442_c2_g1)], peroxidase activity [*CoPOD* (42101_c0_g1)], and xylem development [*CoPRX52* (29405_c1_g1)] (Fig. 7C, D, Additional file 10: Table S9). *CoXTH7* (30876_c0_g5) can catalyze xyloglucan endohydrolysis (XEH) and/or endotransglycosylation (XET). It may be an essential constituent of the primary cell wall that participates in cell wall biogenesis. *CoNPF3.1* (40839_c0_g1) is annotated into the nitrate transporter1/peptide transporter (NPF) family, and it has been described as transmembrane transporter, gibberellic acid homeostasis, and nitrate assimilation in *Arabidopsis thaliana* [21]*.* It may improve the development of buds and the growth of roots [22].

### Validation of the expression of DEGs by qRT-PCR

**Fig. 8:**
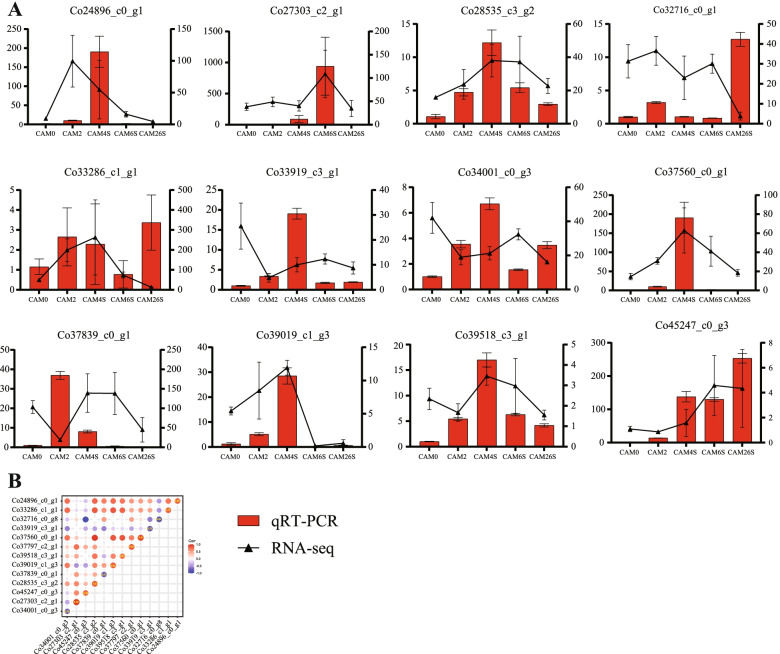
Correlation between expression profiles of selected genes obtained from RNA-seq and qRT-PCR analysis. **A** Relative expressions of qRT-PCR calculated using *Tublin* and *GADPH* as the reference gene is shown in the left *y*-axis. RNA-seq expression of the unigenes is shown in the right *y*-axis. **B** Heatmaps represent expression profiles of selected genes obtained from RNA-seq (left) and qRT-PCR (bottom) analysis. The color scale at the bottom represents Z-score. The values between the two heatmaps represent the correlation value between the expression profiles obtained from RNA-seq and qRT-PCR analysis for each gene. The correlation values above 0.70 are highlighted in bold

To validate the reliability expression profiling obtained by RNA-seq, 12 DEGs with different expression patterns in cotyledons present in the seed were selected for qRT-PCR analysis. The genes chosen for qRT-PCR analysis included 9 TFs and 3 signaling-related hormones genes. For all these genes, the results of qRT-PCR exhibited almost similar expression patterns as compared to those in the RNA-seq data (Fig. 8A), confirming a high correlation between the RN-seq and qRT-PCR data (Fig. 8B).

## Discussion

### Cotyledons play a critical role in regulating seed germination and morphogenesis

Seed germination refers to some biological processes, including the radicle prominence of the seed coat and the resumption of embryo growth, accompanied by nutrient metabolism, DNA repair, and transcription [23, 24]. Cotyledon and endosperm, the primary nutrient storage site, play an important role during seed germination. In *Arabidopsis*, the transcriptomes of the endosperm and embryo were quite similar. Thus, the two tissues are likely to express similar genetic programs that are intrinsic to the seed [25, 26]. It can be assumed that genes expressed in cotyledons are similar to those in embryos, and cotyledons act as a critical partner in seed germination and subsequent growth.

This study characterized the gene expression profiling in the cotyledon of *C.oleifera* in different stages during seed germination. Several critical pathways in seed germination were obtained by KEGG annotation analysis. These pathways are principally involved in cell wall biosynthesis, cell proliferation, primary metabolism, and hormone signaling (Additional file 1: Fig. S4). Similar to the process of seed germination involved the transformation of nutrient metabolism and photosynthesis in other species, many DEGs were identified to be involved in various metabolic pathways. Most of the DEGs were related to the regulation of gene expression, followed by energy production and metastasis (Additional file 1: Fig. S4). These reflect the drastic physiological and genetic regulation activities in the cotyledons, the nutrient storage sources. These results suggested that a certain percent of genes in cotyledons might play largely overlapping roles in governing the seed germination and the seedling emergence of *C.oleifera*, compared with the whole gene set throughout the seed development.

### Candidate hormone associated with *C.oleifera* seed germination

In the present study, many genes were highly expressed in CAM26S, including *GASA3*, *GASA9 GASA11* and *OS08G0141300*. One particular example is the gibberellin-regulated protein that may function in hormonal development control, such as seed germination, flowering, and seed maturation [27]. Meantime, there were many small auxin up RNA (SAUR) genes highly expressed in CAM26S, including *CoSAUR32*, *CoSAUR36*, *CoSAUR55*, *CoSAUR59,* and *CoSAUR74*. The genes of the SAUR family may not only play a central role in auxin-induced acid growth by regulating cell wall acidification but also act independently of auxin.Tissue is specifically regulated by various other hormone pathways and transcription factors [28]. Molecular genetic studies in *Arabidopsis* have revealed the functions of several SAUR genes, such as *AtSARU32*, *AtSAUR36* in cell elongation [29]. Furthermore, some SAURs may perform roles in processes other than cell elongation, such as leaf senescence or cell division. Besides, The *SAUR55*, presented in our data (Fig. 4), was previously reported to be able to encode auxin response protein important for plant growth [30].

Distinct stage-specific expression patterns were observed that most of the identified genes were related to AUXIN, GA, and ETH. They were relatively highly expressed in CAM26S compared with the genes related to KARs and BR, which were highly expressed at CAM0 and CAM2 stages **(**Fig. 3**)**. This suggests the potential roles of AUXIN, GA, and ETH in seedling growth. Our data showed that multiple phytohormone-related genes in cotyledons were differentially reprogrammed during germination, indicating that, at least in part, multiple hormone-related factors in cotyledons synergistically contribute to seed germination and subsequent vegetative growth of *C.oleifera*. The cotyledons are not only the primary nutrient provider but also one of the leading instructors for seed germination and seedling growth.

### Candidate TFs associated with *C.oleifera* seed germination

TFs play important roles in various plant development processes. There were encoded more than 1500 TFs of over 5% of the genome in *Arabidopsis* [31]. It has found about 60 kinds of TF families in higher plants [32], which were involved in regulations of various processes. In the present study, our transcriptome profiling revealed a subset of TFs belonging to AP2/ERF, bHLH, MYB, WRKY, C2H2, C3H, NAC, GRAS, and HB-HD-ZIP families were predominantly expressed in *Camellia* seed germination (Additional file 1: Fig. S5). In CAM2, soaking seeds made the sealed seed embryo and cotyledon exchange substance and energy with the outside world and enter into an active metabolic state, which could prompt the seeds to enter the germination stage from the dormant state. Since ABA and GA act as the main plant hormones regulating seed dormancy and germination, the balance of their biosynthesis and catabolism are essential for ensuring the stability of seed dormancy and germination. It has been found in this research that *CobHLH093*(38274_c0_g1) as a transcription factor regulating gibberellin biosynthesis, *CoAREB3*(37687_c2_g6), and *MYB3*(Co31728_c1_g5) as transcription factors promoting seed dormancy and ABA activation, were highly expressed in the CAM2 stage (Fig. 5). This suggests the synergistic effect of two hormone-related genes. In addition, high expression levels of *CoWOX11*(33796_c4_g1), *CoSPT* (32638_c0_g1), *CoKNAT1*(37948_c2_g1), *CoTINY2*(32839_ c0_g2), *CoSTM* (43196_c1_g1), and *CoPLT2*(38998_c0_g2) (Fig. 5), genes regulating growth of the stem or root in meristem tissues, were found at this stage. Meantime, *CoSGR5*(30478_c0_g2) and *CoUNE10*(44750_c2_g2)*,* which are involved in root gravity, were also highly expressed. Based on this, the switch of seed germination was triggered during imbibition.

## Conclusions

In this study, a de novo assembly of transcriptome data from five stages of *C.oleifera* was performed to provide preliminary insights into the change of cotyledons during seed germination. Enrichment analysis revealed that DEGs were mainly involved in hormone signal transduction and starch sucrose metabolism pathways. The gravitropism regulator *UNE10*, the meristem regulator *STM*, *KNAT1*, *PLT2*, and root-specific transcription factor *WOX11* have higher expression levels in the CAM2 stage (seed soaking). The results indicate that the cotyledon-regulated program for germination had initiated to establish when the seeds were imbibition. The cotyledons play vital roles as the primary nutrient provider and one of the main instructors for seed germination and seedling growth. Taken together, our study will enrich genomic resources of *Camellia* and lay a foundation for further research on molecular mechanisms of development in seed germination and seedling growth of *C.oleifera.*

## Methods

### Plant materials

CL18, as a variety granted number: S-SC-CO-007–2008 in *C.oleifera*, was obtained from the Research Institute of Subtropical Forestry, China Academy of Forestry. Characteristics of CL18 were described by He et al. [33]. Mature seeds of the CL18 variety were collected from Dongfanghong Forestry, Jinhua City, Zhejiang Province, and stored at 4℃ for 2 months. Matured seeds of the CL18 variety were collected from Dongfanghong Forestry Farm, Jinghua City, Zhejiang Province, China, and stored at 4℃ for 2 months. Then, in dark conditions at 25℃, seeds were flooded using deionized water for 2 days, followed by sand stratification in a germination box. After the seeds cracked open, vernier calipers were used to measure the total length of root and stem after germination. By quickly removing the seed coat and the embryo, we collected cotyledon tissues of seeds that were stored at 4℃ (CAM0), soaked in purified water (CAM2), and broken in shell (CAM4S). The cotyledon tissues of seedlings had root growth of 2 cm (CAM6S) or 10 cm (CAM26S) in sand stratification (Fig. 1). The samples were immediately frozen in liquid nitrogen and stored at -80℃ for subsequent RNA-seq or hormone determination. Each test consisted of three biological replicates. The collection of all the samples complies with institutional, national, or international guidelines and legislation. The local forestry management department authorizes the collection of all samples for this research.

### Hormone determination

The content of hormones was determined using liquid chromatography-mass spectrometry (LC–MS) method [13, 34]. Approximately 0.5 g of cotyledon tissues were ground with liquid nitrogen and then suspended in 30 ml of 80% methanol at 4℃ for 24 h in darkness. The extraction mixture was centrifuged at 3500 r /min for 10 min, and the supernatant was collected. After centrifuging, the precipitation was re-suspended in 20 ml of 80% methanol at 4℃ for 1 h. The supernatant merged and evaporated at 40℃ on the rotary evaporator until there was no methanol residual. The remaining water was extracted and decolorized twice with 30 ml of petroleum ether, and the ether was discarded. The aqueous-phase pH was adjusted to 2.9, and 30 ml of ethyl acetate was used to extract the solution 3 times. Then the ester phase was combined, and the solution was decompressed and dried at 40℃. The dry powder was dissolved in 2 ml methanol, filtered with 0.45 m microporous membranes, and stored in a refrigerator at 4℃. Chromatographic column: Hypersil BDS C18 chromatographic column; methanol–water (containing 0.75% glacial acetic acid, 35:65, V/V) was used in the mobile phase. Detection wavelength: 254 nm; Flow rate: 0.8 ml /min; Injection volume 10 ul; Column temperature: 30℃.

### RNA isolation and illumina sequencing of the transcriptome

Approximately 100 mg of fresh cotyledon tissue was used for total RNA extraction, according to the manufacturer's protocol of Trizol reagent (Invitrogen, CA, USA). The total RNA quantity and purity were analyzed using Bioanalyzer 2100 and RNA 6000 Nano LabChip Kit (Agilent, CA, USA) with RIN number > 7.0.

Approximately 10 ug of total RNA was subjected to a isolation from poly(A) mRNA by poly-T oligo attached to magnetic beads (Invitrogen). Following purification, the poly(A)- or poly(A) + RNA fractions were fragmented into small pieces using divalent cations under elevated temperatures. Then the cleaved RNA fragments were reverse-transcribed to create the final cDNA library in accordance with the protocol for the Illumina TruSeq Stranded mRNA Library Prep kit (Illumina, San Diego, USA). The average insert size for the paired-end libraries was 300 bp (± 50 bp). And then, the paired-end sequencing was performed on an Illumina platform (Hiseq 4000) at the LC-BIO (Hangzhou, China), following the vendor's recommended protocol.

### De novo assembly and functional annotation

The quality of raw reads was controlled using Cutadapt and Perl scripts in house, and the sequence quality was further verified using FastQC. De novo assembly of the transcriptome was performed with Trinity 2.4.0 [35]. Trinity grouped transcripts into clusters based on shared sequence content, and the longest transcript in the cluster was selected as the representative gene (aka Unigene). For functional annotation, the unigenes were aligned against the non-redundant (NR) protein database (http://www.ncbi.nlm.nih.gov/), Gene Ontology (GO), SwissProt, Kyoto Encyclopedia of Genes and Genomes (KEGG), and eggNOG databases using DIAMOND with a threshold of E-value < 0.00001[36].

### Identification of differentially expressed genes (DEG)

Salmon [37] was used to perform expression levels for Unigenes by calculating TPM (Transcripts Per kilobase Million) [38]. The DEGs were specified with a threshold of ∣log2(FC)∣ at ≥ 1, *p-*value < 0.01, using edgeR [39].

### Heatmap plotting of DEGs

Heatmap was generated with Z-score normalized TPM values of the DEGs using the online OmicShare tools (https://www.omicstudio.cn/analysis).

### GO and KEGG enrichment analysis

The GO and KEGG pathway enrichment analyses of DEGs were implemented by GOseq R packages [40] and KOBAS software [41], respectively. Both analyses were tested at a significance cutoff FDR ≤ 0.05.

### Identification of transcription factor

The TF families were identified by BLASTX against known plant TFs identified in the iTAK database (http://itak.feilab.net/cgi-bin/itak/index.cgi, version: v1.7) with E-value threshold ≤ 10^–5^.

### Weight gene co-expression network analysis

The input data for the WGCNA were normalized values for each transcript. The DEGs co-expression network was constructed using R package WGCNA(V1.6) with a soft threshold chosen to create networks with a scale-free topology in a way as described by Langfelder and Horvath [42, 43]. After the networks were built, modules of transcripts with similar expression patterns were created, and eigengenes for these modules were calculated. Finally, correlations between these eigengenes and the content of hormones as well as length of seedling were calculated (Additional file 12: Table S11).

### qRT-PCR verification of gene expression

The RNA samples used for qRT-PCR analysis were identical to those for the next-generation sequencing experiments. Single-strand cDNA for each sample was synthesized using the First-Strand Synthesis System (Invitrogen, Carlsbad, CA, USA)0.12 representative DEGs were selected to validate RNA-Seq analysis using qRT-PCR. The Tublin α-3 (*TUB* α*-3*) gene and Glyceraldehyde-3-phosphate dehydrogenase (*GADPH*) was used as the reference gene [44]. The primer pairs were designed according to the selected unigene sequences using Primer 3.0 Plus (http://primer3plus.com/cgi-bin/dev/primer3plus.cgi). The primers were 19–21 bp in length and had amplicon lengths of 200–260 bp (Additional file 13: Table S12). Each qRT-PCR was represented by three biological and three technical replicates. Relative transcript abundance was obtained using the 2^−ΔΔCT^ method [45, 46]. All reactions were carried out in 96-well plate in QuantStudio™ Real-Time PCR Software (Thermo Fisher) with the TB Green® Premix Ex Taq™ (Tli RNaseH Plus)(TaKaRa) kit. The amplification procedure is 95 ℃ for 30 s, followed by 40 cycles of 95℃ for 5 s and 60℃ for 30 s.

## Supplementary Information

**Additional file 1: Fig. S1.** Species distribution of top BLAST hits for matched unigenes sequences. Percentage of unigenes matching the top nine species using Blastx in the NR database. **Fig. S2.** Principal component analysis (PCA) of all samples. Each color on the right indicates the meaning of legend. **Fig. S3.** Histogram presentation of GO classification in different genes. 6820 DEGs were matched by GO terms of three categories. Biological process (blue), Cellular component (green), Molecular function (red). **Fig. S4.** Classification of different genes in the KEGG pathway. The X-axis represents the value of rich factors (the ratio of annotated DEGs to all genes of the enriched pathway). The Y-axis represents the names of pathways. The color depth of each point represents q value. The size of each point represents the number of DEGs. (A) KEGG annotation of DEGs of CAM2 vs. CAM0. (B) KEGG annotation of DEGs of CAM4S vs. CAM2. (C) KEGG annotation of DEGs of CAM6S vs. CAM4S. (D) KEGG annotation of DEGs of CAM26S vs. CAM6S. (E) KEGG annotation of DEGs of CAM6S vs. CAM0. (F) KEGG annotation of DEGs of CAM4S vs. CAM0. (G) KEGG annotation of DEGs of CAM26S vs. CAM0. **Fig. S5.** The differential expression of TFs across five stages. The differential expression of TFs was depicted in heatmap based on Z-score normalized TPM values. The green color indicates lower expressed genes, while the red indicates higher expressed genes.

**Additional file 2: Table S1.** Sequencing statistics of the transcriptome from five stages.

**Additional file 3. Table S2.** Statistics of sample sequencing data evaluation and assembly results.

**Additional file 4: Table S3.** Annotation statistics of unigenes in publicly available databases.

**Additional file 5: Table S4.** The functional annotation of in unigenes.

**Additional file 6: Table S5.** The annotation in the eggNOG database.

**Additional file 7: Table S6.** The different genes in each comparison.

**Additional file 8: Table S7.** The functional annotation of common expression genes in the Venn diagram.

**Additional file 9: Table S8.** The functional annotation of GO and KEGG in all different genes.

**Additional file 10: Table S9.** The functional annotation of all modules in WGCNA.

**Additional file 11: Table S10.** The function of 50 most highly connected hub genes in the MEyellow module.

**Additional file 12: Table S11.** The content of hormone in seeds of C.oleifera.

**Additional file 13: Table S12.** Primer sequence used in qRT-PCR analysis.

## Data Availability

All used sequencing data are available from the NCBI Sequence Read Archive (SRA) database with accession number SRP349608. The transcriptome assembly data is available from the NCBI Sequence Read Archive (SRA) database with accession number PRJNA824722.
