# Supplementary material for: De novo transcriptome assembly of the cotyledon of Camellia oleifera for discovery of genes regulating seed germination
Source: BMC Plant Biol. 2022 May 28;22:265. doi: 10.1186/s12870-022-03651-4 (PMC9145465; doi:10.1186/s12870-022-03651-4)
Supplement: Supplementary file 1 — Additional file 1: Fig. S1. Species distribution of top BLAST hits for matched unigenes sequences. Percentage of unigenes matching the top nine species using Blastx in the NR database. Fig. S2. Principal component analysis (PCA) of all samples. Each color on the right indicates the meaning of legend. Fig. S3. Histogram presentation of GO classification in different genes. 6820 DEGs were matched by GO terms of three categories. Biological process (blue), Cellular component (green), Molecular function (red). Fig. S4. Classification of different genes in the KEGG pathway. The X-axis represents the value of rich factors (the ratio of annotated DEGs to all genes of the enriched pathway). The Y-axis represents the names of pathways. The color depth of each point represents q value. The size of each point represents the number of DEGs. (A) KEGG annotation of DEGs of CAM2 vs. CAM0. (B) KEGG annotation of DEGs of CAM4S vs. CAM2. (C) KEGG annotation of DEGs of CAM6S vs. CAM4S. (D) KEGG annotation of DEGs of CAM26S vs. CAM6S. (E) KEGG annotation of DEGs of CAM6S vs. CAM0. (F) KEGG annotation of DEGs of CAM4S vs. CAM0. (G) KEGG annotation of DEGs of CAM26S vs. CAM0. Fig. S5. The differential expression of TFs across five stages. The differential expression of TFs was depicted in heatmap based on Z-score normalized TPM values. The green color indicates lower expressed genes, while the red indicates higher expressed genes. [file 12870_2022_3651_MOESM1_ESM.zip › Fig.S3 Histogram presentation of GO classification in different genes.pdf]

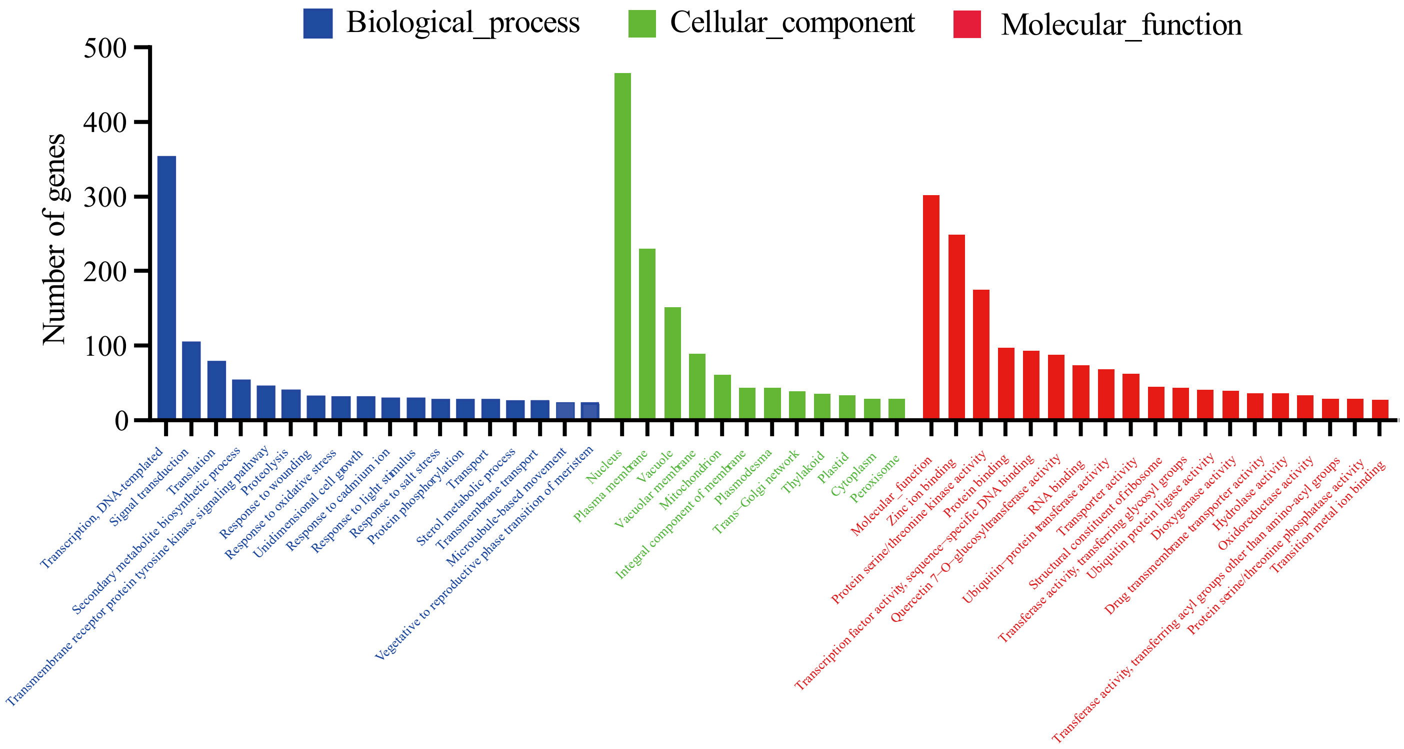

Fig. S3 | Histogram presentation of GO classification in different genes. 6820 DEGs were matched by GO terms of three categories. Biological process (blue), Cellular component (green), Molecular function (red).
